# Multilayer perceptron deep learning radiomics model based on Gd-BOPTA MRI to identify vessels encapsulating tumor clusters in hepatocellular carcinoma: a multi-center study

**DOI:** 10.1186/s40644-025-00895-9

**Published:** 2025-07-07

**Authors:** Mengting Gu, Wenjie Zou, Huilin Chen, Ruilin He, Xingyu Zhao, Ningyang Jia, Wanmin Liu, Peijun Wang

**Affiliations:** 1 Department of Radiology, Tongji Hospital, School of Medicine, Tongji University, Shanghai, China; 2 School of Health Science and Engineering, University of Shanghai for Science and Technology, Shanghai, China; 3 Department of Radiology, Eastern Hepatobiliary Surgery Hospital, The Third Affiliated Hospital of Naval Medical University, Shanghai, China; 4 Department of Radiology, Ruijin Hospital, Shanghai Jiao Tong University School of Medicine, Shanghai, China

## Abstract

**Objectives:**

The purpose of this study is to mainly develop a predictive model based on clinicoradiological and radiomics features from preoperative gadobenate-enhanced (Gd-BOPTA) magnetic resonance imaging (MRI) using multilayer perceptron (MLP) deep learning to predict vessels encapsulating tumor clusters (VETC) in hepatocellular carcinoma (HCC) patients.

**Methods:**

A total of 230 patients with histopathologically confirmed HCC who underwent preoperative Gd-BOPTA MRI before hepatectomy were retrospectively enrolled from three hospitals (144, 54, and 32 in training, test, and validation set, respectively). Univariate and multivariate logistic regression analyses were used to determine independent clinicoradiological predictors significantly associated with VETC, which then constituted the clinicoradiological model. Regions of interest (ROIs) included four modes, intratumoral (Tumor), peritumoral area ≤ 2 mm (Peri2mm), intratumoral + peritumoral area ≤ 2 mm (Tumor + Peri2mm) and intratumoral integrated with peritumoral ≤ 2 mm as a whole (TumorPeri2mm). A total of 7322 radiomics features were extracted respectively for ROI(Tumor), ROI(Peri2mm), ROI(TumorPeri2mm) and 14644 radiomics features for ROI(Tumor + Peri2mm). Least absolute shrinkage and selection operator (LASSO) and univariate logistic regression analysis were used to select the important features. Seven different machine learning classifiers respectively combined the radiomics signatures selected from four ROIs to constitute different models, and compare the performance between them in three sets and then select the optimal combination to become the radiomics model we need. Then a radiomics score (rad-score) was generated, which combined significant clinicoradiological predictors to constituted the fusion model through multivariate logistic regression analysis. After comparing the performance of the three models using area under receiver operating characteristic curve (AUC), integrated discrimination index (IDI) and net reclassification index (NRI), choose the optimal predictive model for VETC prediction.

**Result:**

Arterial peritumoral enhancement and peritumoral hypointensity on hepatobiliary phase (HBP) were independent risk factors for VETC, and constituted the Radiology model, without any clinical variables. Arterial peritumoral enhancement defined as the enhancement outside the tumor boundary in the late stage of arterial phase or early stage of portal phase, extensive contact with the tumor edge, which becomes isointense during the DP. MLP deep learning algorithm integrated radiomics features selected from ROI TumorPeri2mm was the best combination, which constituted the radiomics model (MLP model). A MLP score (MLP_score) was calculated then, which combining the two radiology features composed the fusion model (Radiology MLP model), with AUCs of 0.871, 0.894, 0.918 in the training, test and validation sets. Compared with the two models aforementioned, the Radiology MLP model demonstrated a 33.4%-131.3% improvement in NRI and a 9.3%-50% improvement in IDI, showing better discrimination, calibration and clinical usefulness in three sets, which was selected as the optimal predictive model.

**Conclusion:**

We mainly developed a fusion model (Radiology MLP model) that integrated radiology and radiomics features using MLP deep learning algorithm to predict vessels encapsulating tumor clusters (VETC) in hepatocellular carcinoma (HCC) patients, which yield an incremental value over the radiology and the MLP model.

**Supplementary Information:**

The online version contains supplementary material available at 10.1186/s40644-025-00895-9.

## Introduction

Hepatocellular carcinoma (HCC) accounts for the majority of primary liver malignancies, which were the sixth most prevalent cancer and the third leading cause of cancer-related deaths worldwide [1, 2]. Curative hepatic resection is accepted as the first-line treatment option for patients with well-preserved liver function. However, the high recurrence rate is still the predominant cause of poor overall survival, with 5-year recurrence rates reaching approximately 70% after surgery [3–5].

Vessels encapsulating tumor cluster (VETC) has been recently reported as a novel vascular pattern which seriously influence patient prognosis and it is significantly associated with increased metastasis and recurrence of HCC. VETC is characterized by the presence of CD34 + vessels completely encapsulating tumor clusters (VETC) under the microscope [6–8]. What’s more, VETC pattern can guide therapeutic strategies by predicting treatment response not only to systemic therapies (e.g., sorafenib) but also to intravascular procedures (e.g., transarterial chemoembolization) and percutaneous interventions (e.g., ablation) [9, 10]. Unfortunately, the current diagnosis of VETC relies solely on histopathological examination of resected specimens. Hence, there is an urgent need to predict VETC preoperatively for HCC patients.

However, most studies either identified traditional clinicoradiological characteristics to predict VETC or selected radiomics features for VETC prediction at present [11]. And in most radiomics researches, they predominantly focused on the tumor itself, neglecting the peritumoral area [12]. When radiomics studies involved with the peritumoral areas, they mostly treat the tumor and peritumoral area as two separate Regions of interest (ROIs) to extract features [13]. Consequently, in our study, we consider the intratumoral and peritumoral area as a whole ROI to extract radiomics features, besides, we separately extracted intratumoral and peritumoral features, and select the optimal ROI mode after comparisons. Also, different from traditional machine learning models in most studies, we developed the radiomics model using the multilayer perceptron (MLP) deep learning algorithm and make a comparison between them.

As we all known, MLP is one of the most important forms of artificial neural network (ANN) model that has several applications, such as pattern recognition and so on. It is made up of input layer, multiple hidden layers, and an output layer [14]. MLP is capable of learning complex relationships between inputs and outputs, and can be trained using various algorithms such as backpropagation, which can adjust the weights of the network until the error between the network-generated and the actual output reaches a minimum [15, 16]. Recently, MLP have also been found to be excellent in extracting global feature information [17].

While gadoxetic acid (Gd-EOB-DTPA) is often preferred for hepatobiliary-phase imaging due to its higher hepatocellular uptake (50%) and shorter biliary excretion time (20 min vs. 1 h for Gd-BOPTA), Gd-BOPTA remains widely used for dual-contrast properties [18–20]. This study addresses the clinical need to validate VETC-predictive features using Gd-BOPTA. Compared with traditional extracellular contrast enhanced MR and gadoxetic acid enhanced MR, Gd-BOPTA MR has hepatobiliary phase (HBP) and a real delayed phase (DP), rather than a transitional phase (TP).

In this study, we mainly extracted radiomics features from four ROI modes using seven different machine learnings to constitute different models and compared the performance between them. Then, we proposed a fusion model that integrated clinicoradiological and radiomics model with the best performance of them based on Gd-BOPTA-enhanced MRI for VETC prediction.

## Materials and methods

### Patients

The institutional review boards of three centers approved this retrospective study and waived patient informed consent.

**Fig. 1:**
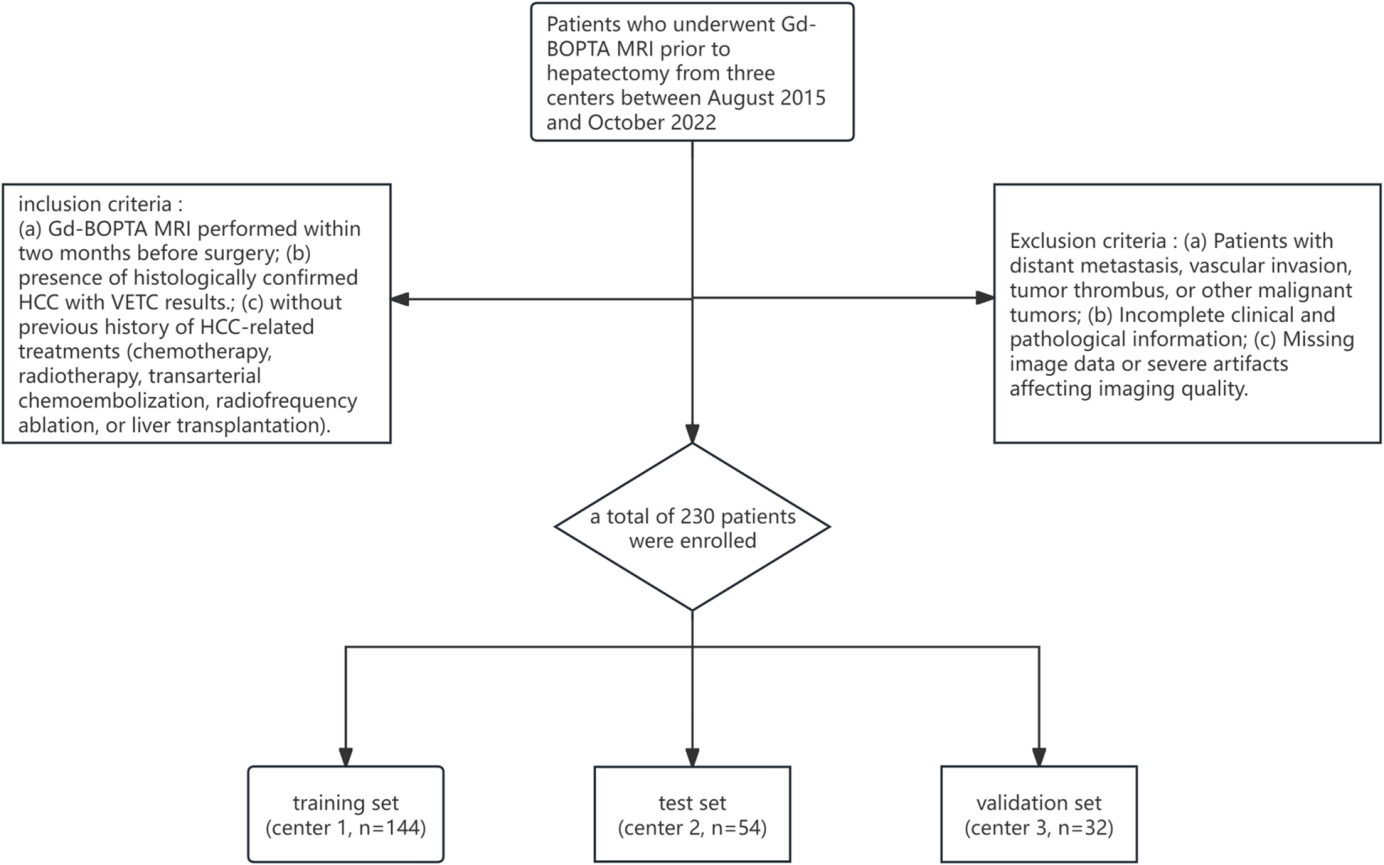
Flowchart shows the patient selection process

Subsequently, a total of 230 patients with histopathologically confirmed HCC who underwent preoperative gadobenate-enhanced (Gd-BOPTA) MRI before hepatectomy were retrospectively included. Among them, 144 patients from center 1 were identified as the training set, 54 patients from center 2 were identified as the test set, and the validation set comprised 32 patients from center 3. The patient selection process is illustrated in Fig. 1.

Patients who underwent Gd-BOPTA MRI prior to hepatectomy from three centers between August 2015 and October 2022 were retrospectively recruited. The inclusion criteria were as follows: (a) Gd-BOPTA MRI performed within two months before surgery; (b) presence of histologically confirmed HCC with VETC results.; (c) without previous history of HCC-related treatments (chemotherapy, radiotherapy, transarterial chemoembolization, radiofrequency ablation, or liver transplantation). Exclusion criteria included: (a) Patients with distant metastasis, vascular invasion, tumor thrombus, or other malignant tumors; (b) Incomplete clinical and pathological information; (c) Missing image data or severe artifacts affecting imaging quality.

### Laboratory examinations and histopathology

The baseline clinical characteristics were retrospectively collected from medical records of three hospitals. Two pathologists with over 10 years of experience in liver pathological analysis independently evaluated all available histological specimens. They were blinded to other information of the patients. The VETC pattern is defined as a continuous lining of CD34 positive endothelium that encapsulate individual tumor clusters, forming a cobweb-like pattern [6–8]. The VETC region was assessed semi-quantitatively on a scale in 5% increments [21]. VETC pattern appearing on 55% or more of the tumor surface (ranging from 0%−100%) was defined as VETC positive (VETC +) [22]. In case of disagreement, consensus was reached through discussion.

### MR image acquisition

Before the scan, patients fasted for 4–6 h and then received an intravenous bolus injection of Gd-BOPTA at a dose of 0.1 mmol/kg, administered at a rate of 2.0 mL/s, followed by a 20-mL saline flush. T1-weighted image (T1WI), T2-weighted image(T2WI) and diffusion-weighted image (DWI) were collected. Arterial, portal, delayed, and hepatobiliary phases (AP, PVP, DP, HBP) were obtained at 20–30 s, 50–60 s, 90–120 s, and 60 min after the injection of GD-BOPTA, respectively. Detailed scanner and scan parameters for the three centers are summarized in Supplementary Table S1-3.

### Radiology feature analysis and clinicoradiological risk factor

MR images were reviewed independently by two abdominal radiologists with 8 and 10 years of MR imaging experience, respectively. If there was any disagreement, a consensus was reached after discussion. The interobserver agreement for qualitative features was evaluated using the kappa test and variables with K coefficient of ≤ 0.85 were removed. The two radiologists independently assessed the following features: (a) tumor diameter, defined as the maximum diameter measured across the largest plane of the tumor, including the capsule [23]; (b) tumor number, classified as solitary (*n* = 1) or multiple (1 < *n* ≤ 3) [24]; (c) tumor margin, categorized as smooth or non-smooth. A smooth margin describes tumors that exhibit a round or oval shape with a continuous and even contour. In contrast, a non-smooth margin signifies tumors with an irregular shape, characterized by uneven or budding portions at the tumor's periphery [25]; (d) shape, round- or oval-like were defined as regular, while others are defined as irregular, such as lobulated, star awn, and needle-like; (e) radiological capsule enhancement, which is a peripheral rim of uniform and smooth hyperenhancement in the PVP or DP, classified into three groups (absent, incomplete, complete) [26]; (f) restricted diffusion, identified by hyperintensity on DWI with a b-value of 600 and hypointensity on ADC [27]; (g) rim arterial phase hyperenhancement (APHE), defined as the irregular ring-like enhancement seen around hypovascular central areas of a tumor during the arterial phase of imaging [28]; (h) Nonrim APHE [29]; (i) Arterial peritumoral enhancement, characterized by a crescent-shaped or polygonal area located outside the tumor margin, which displays hyperintensity during the arterial phase (AP) of imaging and isointensity during the portal venous phase (PVP) [30]; (j) nonperipheral"washout", refers to hypointensity of the lesions compared to the surrounding liver parenchyma in any late phase other than the AP [31]; (k) enhancement pattern, classified as typical dynamic enhancement with arterial hyperintensity and PVP/DP washout, or atypical enhancement [32, 33]. (l) intratumoral necrosis, the area in the tumor without enhancement, high signal on T2WI, low signal on T1WI; (m) tumor hypointensity on HBP, presenting as hypointense tumor on HBP images, when compared with the surrounding liver parenchyma [34]; (n) peritumoral hypointensity on HBP, presenting as hypointense areas of liver parenchyma located outside of the tumor margin in crescent shape, wedge shape, or flame-like shape on HBP images [30, 35].

### Radiomics analysis of MR images

**Fig. 2:**
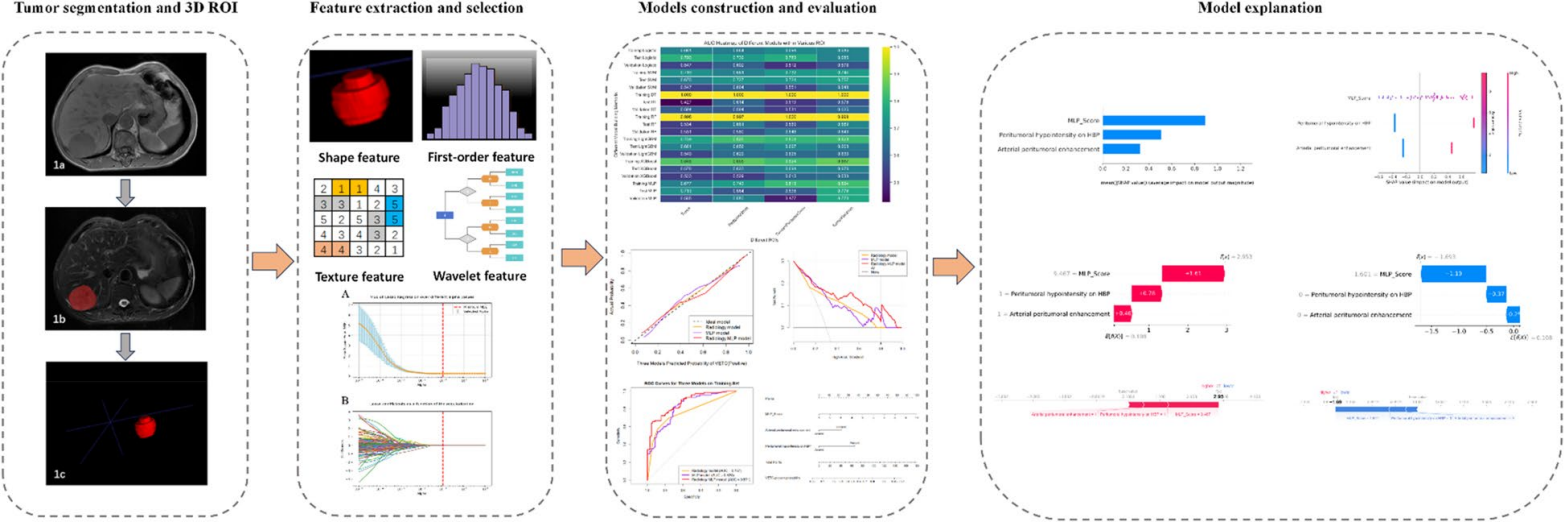
Flowchart of radiomics analysis

Radiomics workflow comprised image segmentation, feature extraction, feature selection, and model construction and evaluation (Fig. 2).

### Image segmentation

The entire tumor segmentation exercise was accomplished by a radiologist (reader A, 8 years of abdominal imaging experiences) with ITK-SNAP software. Regions of interests were manually drawn on the T1-weighted images (T1WI), T2-weighted images(T2WI), diffusion-weighted images (DWI), enhanced arterial phase (AP), portal venous phase (PVP), delayed phase (DP), and HBP (Hepatobiliary phase) images, covering the whole tumor. The intratumoral region was defined as the area within radiologist-annotated tumor boundaries. The peritumoral area ≤ 2 mm was generated by python algorithm based on the intratumoral ROI (Tumor), a portion of the ROI beyond the liver parenchyma was removed manually. Feature post-fusion is the combination of intratumoral and peritumoral area ≤ 2 mm as two separate ROIs (Tumor + Peri2mm). Feature pre-fusion refers to the delineation of intratumoral and peritumoral area ≤ 2 mm as a whole ROI (TumorPeri2mm). A total of 30 patients were randomly selected, then the same procedure repeated by another radiologist (reader B, 10 years of abdominal imaging experiences). Reader A repeated the same procedure one month later. The inter- and intra-class correlation coefficient (ICC) was calculated.

### Radiomics feature extraction and selection

**Fig. 3:**
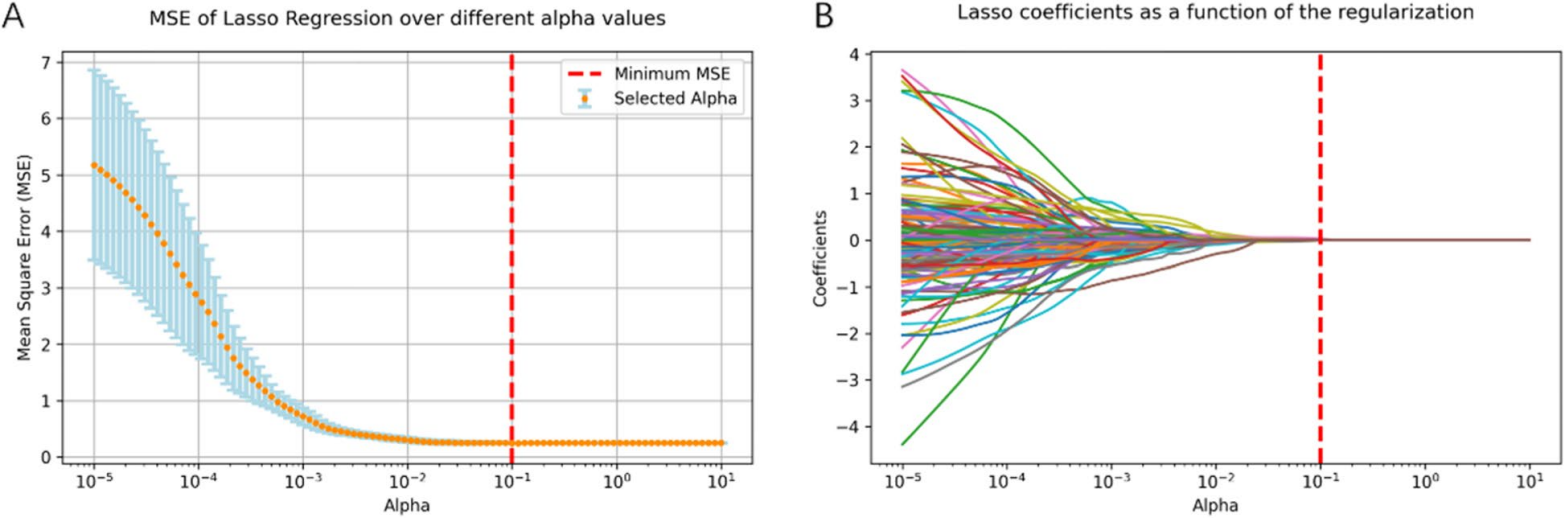
Radiomics feature selection using the LASSO regression algorithm. The fivefold cross-validation process was performed to obtain the optimal tuning parameter Alpha. **A** The optimal alpha value of 10^–1^ for the subsequent analysis. **B** Three features from MRI images with non-zero coefficients were chosen for the radiomics model construction according to the optimal alpha

Radiomic features of four ROI modes were extracted using PyRadiomics. Radiomics features were extracted including first-order features, shape features, texture features, wavelet-transformed and laplacian of gaussian features within four ROIs, which were ROI(Tumor), ROI(Peri2mm), ROI(TumorPeri2mm) and ROI(Tumor + Peri2mm). A set of 1046 radiomic features for each MRI phase (T1WI, T2WI, DWI, AP, PVP, DP, HBP) were extracted for each ROI, which means a total of 7322 radiomics features for ROI(Tumor), ROI(Peri2mm), ROI(TumorPeri2mm) and 14,644 radiomics features for ROI(Tumor + Peri2mm). For each ROI, the radiomics features with ICC less than 0.85 were removed. Then KNN (K-NearestNeighbor) imputation was performed for the remaining radiomics features and then they were standardized into a normal distribution with z-scores to eliminate index dimension differences of the data. The selections of radiomics features under four ROI modes were all as follows. First, the Mann–Whitney *U* or Student *t* test was employed to ascertain the difference between VETC positive(VETC +) and VETC negative(VETC-). The significant variables with *p* < 0.05 were selected. Second, we used univariate logistic regression analysis to select the useful radiomics features. The significant variables with *p* < 0.05 were retained. Third, Spearman’s rank correlation was implemented to eliminate features with a correlation coefficient greater than 0.75. Finally, we applied the least absolute shrinkage and selection operator (LASSO) method, which can reduce the dimension to select the features with the largest amount of information. The Alpha was optimized through the fivefold cross-validation method (Fig. 3).

### Model construction and evaluation

#### Clinicoradiological model construction

First, univariate logistic regression analysis was performed on all clinical variables and MR imaging indicators. Then, features with *P* values less than 0.05 were included in the multivariate logistic regression analysis to identify independent risk factors associated with VETC and construct a clinicoradiological model.

### Radiomics model construction

Machine learning (ML) models were developed by seven different ML classifiers: logistic regression, support vector machine (SVM), decision tree (DT), random forest (RF), Light Gradient Boosting Machine (LightGBM), eXtreme Gradient Boosting (XGBoost) and multilayer perceptron (MLP), which respectively combined the radiomics signatures selected from the four modes of ROIs, and compared the performance between them in three cohorts, respectively. When most of the ML models perform well in three cohorts in that ROI, the ML model with the best performance in corresponding ROI was the ultimate radiomics model.

### Fusion model construction

Subsequently, a radiomics score (Rad_score) was calculated, which combining clinicoradiological features constituted the fusion model using multivariate logistic regression.

### Model evaluations and comparisons

The discrimination performance of the models was measured by area under the receiver operating characteristics (ROC) curve. Calibration curves were employed to evaluate predictive accuracy. And, decision curve analysis (DCA) was performed to assess the clinical utility of the nomograms by quantifying the net benefits across various threshold probabilities. The performance of three models in three sets was compared by AUC, sensitivity, specificity, PPV (positive predictive value), NPV (negative predictive value) and accuracy. The Net Reclassification Improvement (NRI) and Integrated Discrimination Improvement (IDI) were used to evaluate the improvement in model performance. Finally, we select the model with the best performance in predicting VETC as the optimal predictive model, and a nomogram was built as a graphical presentation.

### Statistical analysis

Statistical analyses were performed with IBM SPSS Statistics (version 25.0) and R software (version 4.3.2).

Continuous variables following a normal distribution with homogeneous variance are expressed as mean ± standard deviation (SD) and compared using Student’s t test. While for variables that do not meeting these criteria, which are expressed as median (interquartile range) and compared using the Mann–Whitney U test. Categorical variables were expressed as case numbers (percentages) and compared using the chi-square test. The DeLong test was used to compare the AUCs of these models. All analyses were considered signifcant at *p* values of less than 0.05 (two-tailed).

SHapley Additive Explanations (SHAP) is a method for interpreting the results of predictive models based on cooperative game theory [36], which involved both global and local strategies. At the global level, two primary visualizations are leveraged: a summary bee swarm plot and a bar graph depicting the average SHAP values for each feature. The bee swarm plot offers a panoramic view of feature significance, illustrating how each feature influences predictions across the entire dataset. Similarly, the bar chart underscores the importance of key features that significantly drive the model's outcomes. For local interpretation, waterfall plots and force plots were used to explain predictions on an individual basis. Waterfall plots are utilized to visually represent how each individual feature contributes to a specific prediction. Meanwhile, force plots offer a more intricate and granular view, displaying the exact impact of each feature on the predicted outcome for a single patient.

## Result

### Clinicoradiological characteristics

Two hundred thirty patients were totally collected from three hospitals, including 144 patients in the training set, 44 patients in the test set and 32 patients in the validation set. Among all 230 patients, VETC was pathologically diagnosed in 117 patients. The incidences of VETC positive were 50.7%, 51.9%, 50% in the training set (*n* = 144, 73 VETC positive and 71 VETC negative), test set (*n* = 54, 28 VETC positive and 26 VETC negative) and validation set (*n* = 32, 16 VETC positive and 16 VETC negative). No significant differences were observed between the three sets. There were 119 male patients (82.6%) and 25 female patients (17.4%) in the training set, 47 male patients (87.0%) and 7 female patients (13.0%) in the test set, and 24 male patients (75.0%) and 8 female patients (25.0%) in the validation set. Baseline characteristics of three cohorts included are summarized in Table 1.

**Table 1:** Baseline characteristics in training, test and validation sets for predicting VETC

Characteristic	Training set (*n* = 144)			Test set (*n* = 54)			Validation set (*n* = 32)		
	**VETC absent (*n* = 71)**	**VETC present (*n* = 73)**	***P* value**	**VETC absent (*n* = 26)**	**VETC present (*n* = 28)**	***P* value**	**VETC absent (*n* = 16)**	**VETC present (*n* = 16)**	***P* value**
**Clinical features**									
Age	56.03 ± 10.04	54.92 ± 11.12	0.531	57.50 (52.50–60.75)	51.50 (44.75–62.00)	0.368	54.62 ± 13.83	60.00 ± 9.59	0.211
Gender									
Male	57 (80.3%)	62 (84.9%)	0.606	23 (88.5%)	24 (85.7%)	1.000	13 (81.2%)	11 (68.8%)	0.683
Female	14 (19.7%)	11 (15.1%)		3 (11.5%)	4 (14.3%)		3 (18.8%)	5 (31.2%)	
BCLC stage									
0	21 (29.6%)	13 (17.8%)	0.242	15 (57.7%)	1 (3.6%)	< 0.001	3 (18.8%)	1 (6.2%)	0.435
A	45 (63.4%)	53 (72.6%)		10 (38.5%)	25 (89.3%)		2 (12.5%)	4 (25.0%)	
B	5 (7.0%)	7 (9.6%)		1 (3.8%)	2 (7.1%)		11 (68.8%)	11 (68.8%)	
Child–Pugh stage									
A	68 (95.8%)	70 (95.9%)	1.000	25 (96.2%)	27 (96.4%)	1.000	14 (87.5%)	12 (75.0%)	0.651
B	3 (4.2%)	3 (4.1%)		1 (3.8%)	1 (3.6%)		2 (12.5%)	4 (25.0%)	
Liver disease									
HBV	65 (91.5%)	61 (83.6%)	0.231	23 (88.5%)	25 (89.3%)	1.000	11 (68.8%)	14 (87.5%)	0.392
None or other	6 (8.5%)	12 (16.4%)		3 (11.5%)	3 (10.7%)		5 (31.2%)	2 (12.5%)	
AFP(ng/L)	31.80 (4.25–181.40)	42.10 (9.40–302.20)	0.160	6.90 (3.52–177.12)	12.15 (3.50–233.62)	0.972	7.80 (3.58–171.22)	71.00 (11.65–1210.00)	0.121
PIVKA-II(mAU/mL)	1.99 (1.52–2.72)	2.29 (1.67–3.12)	0.046	1.98 ± 0.63	2.27 ± 0.90	0.173	2.72 ± 0.99	2.77 ± 1.22	0.899
CA199(U/mL)	14.70 (7.55–23.40)	18.40 (9.90–30.50)	0.088	14.70 (10.15–27.52)	16.05 (9.85–28.50)	0.869	12.55 (7.82–22.10)	23.00 (11.70–39.10)	
CEA(ng/mL)	2.10 (1.40–2.80)	2.50 (1.80–3.20)	0.067	2.35 (1.70–3.62)	2.15 (1.18–4.22)	1.000	2.70 (1.50–4.95)	2.70 (1.85–3.35)	
ALT(U/L)	27.00 (18.50–38.00)	26.00 (19.00–45.00)	0.450	26.00 (19.50–40.25)	30.50 (20.50–39.75)	0.723	26.50 (20.00–43.25)	34.50 (29.50–41.00)	0.439
AST(U/L)	24.00 (18.00–33.50)	30.00 (21.00–40.00)	0.017	23.00 (19.25–32.25)	25.50 (19.75–33.50)	0.516	27.00 (21.50–38.00)	33.00 (24.50–45.00)	0.300
TP(g/L)	68.75 ± 6.40	69.63 ± 6.76	0.425	67.96 ± 5.24	70.04 ± 3.81	0.100	68.85 ± 5.95	69.17 ± 4.16	0.859
ALB(g/L)	41.90 (39.10–44.85)	42.80 (39.90–46.10)	0.255	42.35 (39.95–45.40)	43.40 (41.35–46.10)	0.264	43.60 (39.97–47.43)	41.30 (38.90–43.15)	0.083
GLOB(g/L)	26.40 (24.25–28.65)	26.50 (23.90–30.00)	0.793	25.84 ± 4.04	26.82 ± 2.97	0.311	25.61 ± 3.42	27.94 ± 3.88	0.082
TBIL(μmol/L)	13.90 (11.65–17.90)	14.40 (11.70–19.30)	0.545	14.85 (12.72–18.80)	14.25 (11.35–18.60)	0.945	10.00 (7.88–12.12)	15.15 (11.20–21.20)	0.011
DBIL(μmol/L)	5.40 (4.20–6.90)	5.30 (4.00–7.20)	0.792	5.10 (4.40–6.17)	5.55 (4.20–7.33)	0.494	4.35 (3.60–5.03)	6.20 (4.88–8.33)	0.006
IBIL(μmol/L)	8.70 (7.40–10.85)	8.90 (7.30–12.30)	0.425	8.95 (6.75–11.38)	8.35 (6.67–10.85)	0.965	5.40 (4.40–7.43)	7.50 (6.95–11.43)	0.029
CHE(U/L)	7221.79 ± 1959.35	7262.58 ± 1641.79	0.892	6766.62 ± 1671.46	7504.07 ± 1595.12	0.103	7349.50 (6357.75–7863.5)	6576.00 (5851.00–7292)	
CG(ug/ML)	1.10 (0.50–2.35)	1.30 (0.50–2.60)	0.809	1.20 (0.53–2.15)	0.60 (0.47–1.12)	0.059	1.45 (1.00–1.95)	1.95 (1.42–2.62)	
TBA(μmol/L)	5.50 (3.20–11.85)	6.00 (3.40–11.30)	0.967	4.65 (2.60–11.60)	4.70 (2.52–6.67)	0.203	5.20 (3.35–8.65)	6.45 (4.03–13.43)	0.250
GGT(U/L)	40.00 (26.00–85.00)	45.00 (33.00–78.00)	0.508	43.50 (22.00–65.25)	39.00 (26.75–60.25)	0.856	32.50 (20.50–75.75)	61.00 (30.50–122.00)	0.132
AFU(U/L)	23.00 (19.00–27.50)	24.00 (19.00–30.00)	0.925	20.00 (17.00–25.00)	24.00 (20.00–28.25)	0.074	27.50 (24.00–30.75)	29.00 (23.00–34.00)	
CRP(mg/L)	0.78 (0.50–2.93)	1.51 (0.60–3.86)	0.066	1.47 (0.50–1.88)	0.68 (0.50–2.78)	0.378	5.00 (5.00–5.00)	5.00 (5.00–8.05)	0.075
PLT(10^9/L)	139.00 (102.00–192)	151.00 (117.00–187)	0.643	151.62 ± 59.52	161.75 ± 47.20	0.490	132.50 (121.00–179.75)	129.50 (111.00–165.25)	0.925
PT(S)	12.10 (11.35–12.65)	12.00 (11.50–12.60)	0.792	12.05 ± 0.86	12.00 ± 0.81	0.827	11.30 (11.05–11.95)	12.20 (11.70–12.78)	0.013
APTT(S)	28.40 (25.65–31.35)	28.50 (25.80–31.90)	0.834	29.00 (27.43–32.90)	27.45 (25.48–30.00)	0.087	27.70 (26.18–30.32)	28.55 (26.95–29.85)	0.720
TT(S)	20.20 (19.10–21.05)	20.30 (19.60–20.90)	0.186	20.13 ± 1.38	20.38 ± 1.51	0.532	18.58 ± 0.69	18.42 ± 1.19	0.652
FBG(g/L)	2.07 (1.85–2.63)	2.11 (1.86–2.40)	0.517	2.42 ± 0.78	2.22 ± 0.52	0.258	2.22 ± 0.67	2.71 ± 0.77	0.067
CHOL(mmol/L)	3.85 ± 0.78	4.10 ± 0.74	0.047	4.07 ± 0.90	3.80 ± 0.57	0.190			
TG(mmol/L)	0.97 (0.77–1.26)	1.08 (0.84–1.41)	0.086	1.05 ± 0.38	1.18 ± 0.49	0.307			
HDL-C(mmol/L)	1.19 (0.98–1.38)	1.19 (1.02–1.42)	0.797	1.26 ± 0.34	1.15 ± 0.29	0.206			
LDL-C(mmol/L)	2.37 (1.97–2.69)	2.59 (2.28–3.01)	0.041	2.58 ± 0.83	2.41 ± 0.54	0.361			
HBsAg									
Negative	8 (11.3%)	17 (23.3%)	0.092	3 (11.5%)	3 (10.7%)	1.000	2 (12.5%)	2 (12.5%)	1.000
Positive	63 (88.7%)	56 (76.7%)		23 (88.5%)	25 (89.3%)		14 (87.5%)	14 (87.5%)	
HBsAb									
Negative	61 (85.9%)	63 (86.3%)	1.000	23 (88.5%)	24 (85.7%)	1.000	10 (62.5%)	12 (75.0%)	0.703
Positive	10 (14.1%)	10 (13.7%)		3 (11.5%)	4 (14.3%)		6 (37.5%)	4 (25.0%)	
HBeAg									
Negative	57 (80.3%)	52 (71.2%)	0.284	19 (73.1%)	20 (71.4%)	1.000	14 (87.5%)	12 (75.0%)	0.651
Positive	14 (19.7%)	21 (28.8%)		7 (26.9%)	8 (28.6%)		2 (12.5%)	4 (25.0%)	
HBeAb									
Negative	22 (31.0%)	28 (38.4%)	0.451	10 (38.5%)	12 (42.9%)	0.959	5 (31.2%)	3 (18.8%)	0.683
Positive	49 (69.0%)	45 (61.6%)		16 (61.5%)	16 (57.1%)		11 (68.8%)	13 (81.2%)	
HBcAb									
Negative	1 (1.4%)	4 (5.5%)	0.379	0 (0.0%)	1 (3.6%)	1.000	3 (18.8%)	1 (6.2%)	0.593
Positive	70 (98.6%)	69 (94.5%)		26 (100.0%)	27 (96.4%)		13 (81.2%)	15 (93.8%)	
HBV/C-DNA									
< 50 IU/ml	44 (62.0%)	34 (46.6%)	0.309	7 (26.9%)	9 (32.1%)	0.272	10 (62.5%)	5 (31.2%)	0.267
50–10^3	5 (7.0%)	8 (11.0%)		9 (34.6%)	7 (25.0%)		1 (6.2%)	3 (18.8%)	
10^3–10^5	11 (15.5%)	14 (19.2%)		2 (7.7%)	7 (25.0%)		2 (12.5%)	5 (31.2%)	
> 10^5	11 (15.5%)	17 (23.3%)		8 (30.8%)	5 (17.9%)		3 (18.8%)	3 (18.8%)	
MRI features									
Tumor diameter(cm)	2.90 (2.05–4.25)	3.50 (2.30–5.30)	0.060	2.05 (1.50–3.25)	3.65 (2.88–5.08)	< 0.001	5.19 ± 2.88	6.00 ± 3.66	0.494
Tumor number									
Solitary	66 (93.0%)	63 (86.3%)	0.301	25 (96.2%)	26 (92.9%)	1.000	14 (87.5%)	11 (68.8%)	0.392
Multiple	5 (7.0%)	10 (13.7%)		1 (3.8%)	2 (7.1%)		2 (12.5%)	5 (31.2%)	
Shape									
Regular	47 (66.2%)	37 (50.7%)	0.086	19 (73.1%)	20 (71.4%)	1.000	11 (68.8%)	7 (43.8%)	0.285
Irregular	24 (33.8%)	36 (49.3%)		7 (26.9%)	8 (28.6%)		5 (31.2%)	9 (56.2%)	
Margin									
Smooth	42 (59.2%)	30 (41.1%)	0.045	13 (50.0%)	17 (60.7%)	0.605	8 (50.0%)	5 (31.2%)	0.472
Non-smooth	29 (40.8%)	43 (58.9%)		13 (50.0%)	11 (39.3%)		8 (50.0%)	11 (68.8%)	
Radiological capsule enhancement									
Complete	28 (39.4%)	29 (39.7%)	0.870	11 (42.3%)	10 (35.7%)	0.693	4 (25.0%)	1 (6.2%)	0.247
Incomplete	26 (36.6%)	29 (39.7%)		11 (42.3%)	15 (53.6%)		7 (43.8%)	11 (68.8%)	
Absent	17 (23.9%)	15 (20.5%)		4 (15.4%)	3 (10.7%)		5 (31.2%)	4 (25.0%)	
Restricted diffusion									
Present	67 (94.4%)	68 (93.2%)	1.000	24 (92.3%)	28 (100.0%)	0.439	15 (93.8%)	15 (93.8%)	1.000
Absent	4 (5.6%)	5 (6.8%)		2 (7.7%)	0 (0.0%)		1 (6.2%)	1 (6.2%)	
Nonrim APHE									
Present	43 (60.6%)	43 (58.9%)	0.974	14 (53.8%)	18 (64.3%)	0.615	10 (62.5%)	13 (81.2%)	0.432
Absent	28 (39.4%)	30 (41.1%)		12 (46.2%)	10 (35.7%)		6 (37.5%)	3 (18.8%)	
Rim APHE									
Absent	45 (63.4%)	47 (64.4%)	1.000	19 (73.1%)	19 (67.9%)	0.903	11 (68.8%)	9 (56.2%)	0.715
Present	26 (36.6%)	26 (35.6%)		7 (26.9%)	9 (32.1%)		5 (31.2%)	7 (43.8%)	
Arterial peritumoral enhancement									
Absent	58 (81.7%)	33 (45.2%)	< 0.001	22 (84.6%)	15 (53.6%)	0.031	12 (75.0%)	13 (81.2%)	1.000
Present	13 (18.3%)	40 (54.8%)		4 (15.4%)	13 (46.4%)		4 (25.0%)	3 (18.8%)	
Nonperipheral"washout"									
Present	45 (63.4%)	44 (60.3%)	0.832	12 (46.2%)	18 (64.3%)	0.287	10 (62.5%)	10 (62.5%)	1.000
Absent	26 (36.6%)	29 (39.7%)		14 (53.8%)	10 (35.7%)		6 (37.5%)	6 (37.5%)	
Enhancement pattern									
Typical	45 (63.4%)	42 (57.5%)	0.585	15 (57.7%)	12 (42.9%)	0.414	9 (56.2%)	9 (56.2%)	1.000
Atypical	26 (36.6%)	31 (42.5%)		11 (42.3%)	16 (57.1%)		7 (43.8%)	7 (43.8%)	
Intratumoral necrosis									
Absent	56 (78.9%)	48 (65.8%)	0.116	23 (88.5%)	21 (75.0%)	0.357	12 (75.0%)	9 (56.2%)	0.457
Present	15 (21.1%)	25 (34.2%)		3 (11.5%)	7 (25.0%)		4 (25.0%)	7 (43.8%)	
Tumor hypointensity on HBP									
Atypical	33 (46.5%)	38 (52.1%)	0.615	10 (38.5%)	11 (39.3%)	1.000	4 (25.0%)	5 (31.2%)	1.000
Typical	38 (53.5%)	35 (47.9%)		16 (61.5%)	17 (60.7%)		12 (75.0%)	11 (68.8%)	
Peritumoral hypointensity on HBP									
Absent	63 (88.7%)	31 (42.5%)	< 0.001	23 (88.5%)	13 (46.4%)	0.003	14 (87.5%)	8 (50.0%)	0.057
Present	8 (11.3%)	42 (57.5%)		3 (11.5%)	15 (53.6%)		2 (12.5%)	8 (50.0%)	

*Abbreviations*: *MVI* microvascular invation, *BCLC* Barcelona Clinic Liver Cancer, *HBV* hepatitis B virus, *AFP* alpha-fetoprotein, *PIVKA-II* protein induced by vitamin K absence or antagonist-II, *CA199* carbohydrate antigen 19–9, *CEA* carcinoembryonic antigen, *ALT* alanine aminotransferase, *AST* aspartate aminotransaminase, *TP* total protein, *ALB* albumin, *GLOB* globulin, *TBIL* total bilirubin, *DBIL* direct bilirubin, *IBIL* indirect bilirubin, *CHE* cholinesterase, *CG* glycocholic acid, *TBA* total bile acid, *GGT* r-glutamyltransferase, *AFU* a-fucosidase, *CRP* C-reactive protein, *PLT* platelet count, *PT* prothrombin time, *APTT* activated partial thromboplastin time, *TT* thrombin time, *FBG* fibrinogen, *CHOL* total cholesterol, *TG* triglyceride, *HDL-C* high density lipoprotein cholesterol, *LDL-C* low density lipoprotein cholesterol, *APHE* arterial phase hyperenhancement, *HBP* hepatobiliary phase

### Radiology model construction

PIVKA-II (Lg10), Margin, Arterial peritumoral enhancement and Peritumoral hypointensity on HBP were related to VETC in univariate analysis at a test level of *p* < 0.05. All the above four variables were included in the multivariate logistic regression analysis, which showed that only Arterial peritumoral enhancement (OR 2.440; 95%CI:0.901–6.607) and peritumoral hypointensity on HBP (OR 4.130; 95%CI:1.441–11.841) were independent risk factors for VETC and costruct the Radiology model, without any clinical characteristics. The univariate and multivariate logistic regression results of clinicoradiological characteristics are summarized in Table 2. The radiology model demonstrated AUC values of 0.767 in the training set, 0.756 in the test set and 0.684 in the validation cohort, respectively (Table 3).

**Table 2:** Univariate and multivariate logistic regression analysis in training set for predicting VETC

Variable	Univariable Logistic Regression			Multivariable Logistic Regression		
	**OR**	**95% CI**	***P***	**OR**	**95% CI**	***P***
PIVKA-II (Lg10)	1.543	1.032,2.306	0.035			
Margin (Nonsmooth)	2.076	1.068,4.034	0.031			
Arterial peritumoral enhancement (Present)	5.408	2.535,11.538	< 0.001	2.440	0.901,6.607	0.079
Peritumoral hypointensity on HBP(Present)	10.669	4.471,25.462	< 0.001	4.130	1.441,11.841	0.008
MLP_Score	1.657	1.402,1.960	< 0.001	1.541	1.292,1.838	< 0.001

**Table 3:** Performance of radiology, MLP and radiology MLP model for predicting VETC

	Radiology model			MLP model			Radiology MLP model		
	Training	Test	Validation	Training	Test	Validation	Training	Test	Validation
AUC	0.767	0.756	0.684	0.824	0.776	0.770	0.871	0.894	0.918
Accuracy	0.729	0.704	0.688	0.764	0.778	0.812	0.799	0.852	0.938
f1-score	0.683	0.652	0.615	0.779	0.806	0.750	0.764	0.857	0.913
Sensitivity	0.575	0.536	0.500	0.821	0.893	0.813	0.644	0.857	0.938
Specificity	0.887	0.885	0.875	0.704	0.634	0.750	0.958	0.846	0.875
PPV	0.840	0.833	0.800	0.741	0.735	0.813	0.940	0.857	0.889
NPV	0.670	0.639	0.636	0.794	0.850	0.813	0.723	0.846	0.850

*AUC* area under receiver operating characteristic curve, *PPV* positive predictive value, *NPV* negative predictive value, *MLP *Multi-layer Perceptron

### MLP model construction

For the selected radiomics features within four ROIs, we respectively use seven machine learning algorithms to construct different radiomics models and compare the performances between them (Table 4). The heatmap were used to show the AUCs between the different ML models within various ROIs (Supplementary Fig S1). The majority of these models perform well in three cohorts in ROI (TumorPeri2mm), the best-performing machine learning model was MLP deep learning model, with AUC of 0.824, 0776, 0.770 in the training, test and validation set, respectively. Through training the MLP model, we find that when the two hidden layers were 5 and 3 the MLP model performs the best. Consequently, we choose MLP to construct the ultimate radiomics model. We selected three features which extracted from ROI (TumorPeri2mm), TumorPeri2mm_DP_wavelet-LHL_glcm_MaximumProbability, TumorPeri2mm_HBP_original_gldm_DependenceNonUniformityNormalized and Peri2mm_DWI_wavelet-HLH_firstorder_Kurtosis, to establish the MLP deep learning model. The features of other ROI modes that selected for model construction were shown in the Supplementary Table S4.

**Table 4:** AUC of Different Models within Various ROI

AUC		Tumor	Peri2mm	Tumor + Peri2mm	TumorPeri2mm
Logistic	Training	0.681	0.664	0.694	0.686
	Test	0.793	0.739	0.783	0.685
	Validation	0.547	0.602	0.512	0.578
SVM	Training	0.719	0.681	0.732	0.744
	Test	0.670	0.727	0.724	0.757
	Validation	0.547	0.604	0.551	0.648
DT	Training	1.000	1.000	1.000	1.000
	Test	0.427	0.614	0.519	0.578
	Validation	0.594	0.594	0.531	0.625
RF	Training	0.998	0.997	1.000	0.999
	Test	0.534	0.661	0.569	0.689
	Validation	0.531	0.580	0.646	0.643
LightGBM	Training	0.759	0.826	0.808	0.823
	Test	0.681	0.656	0.697	0.663
	Validation	0.549	0.629	0.625	0.630
XGBoost	Training	0.846	0.855	0.824	0.867
	Test	0.576	0.633	0.694	0.676
	Validation	0.533	0.529	0.613	0.633
MLP	Training	0.677	0.743	0.813	0.824
	Test	0.713	0.584	0.598	0.776
	Validation	0.555	0.680	0.477	0.770

*AUC* area under receiver operating characteristic curve, *SVM* Support Vector Machines, *DT* Decision Tree, *RF* Random Forest, *LightGBM* Light Gradient Boosting Machine, *XGBoost* eXtreme Gradient Boosting, *MLP* Multi-layer Perceptron

### Radiology MLP model construction

Then a MLP score (MLP_score) calculated then, which combining the two radiology features constituted the radiology MLP model, showed the highest predictive performance in the training (AUC = 0.871; sensitivity:0.644; specificity:0.958), test (AUC = 0.894; sensitivity:0.857; specificity:0.846) and validation set (AUC = 0.918; sensitivity:0.938; specificity:0.875) (Table 3,). Heatmap showed that low correlation coefficients were observed in the selected radiology and radiomics features (Supplementary Fig S2).

### Model evaluations and comparisons

**Fig. 4:**
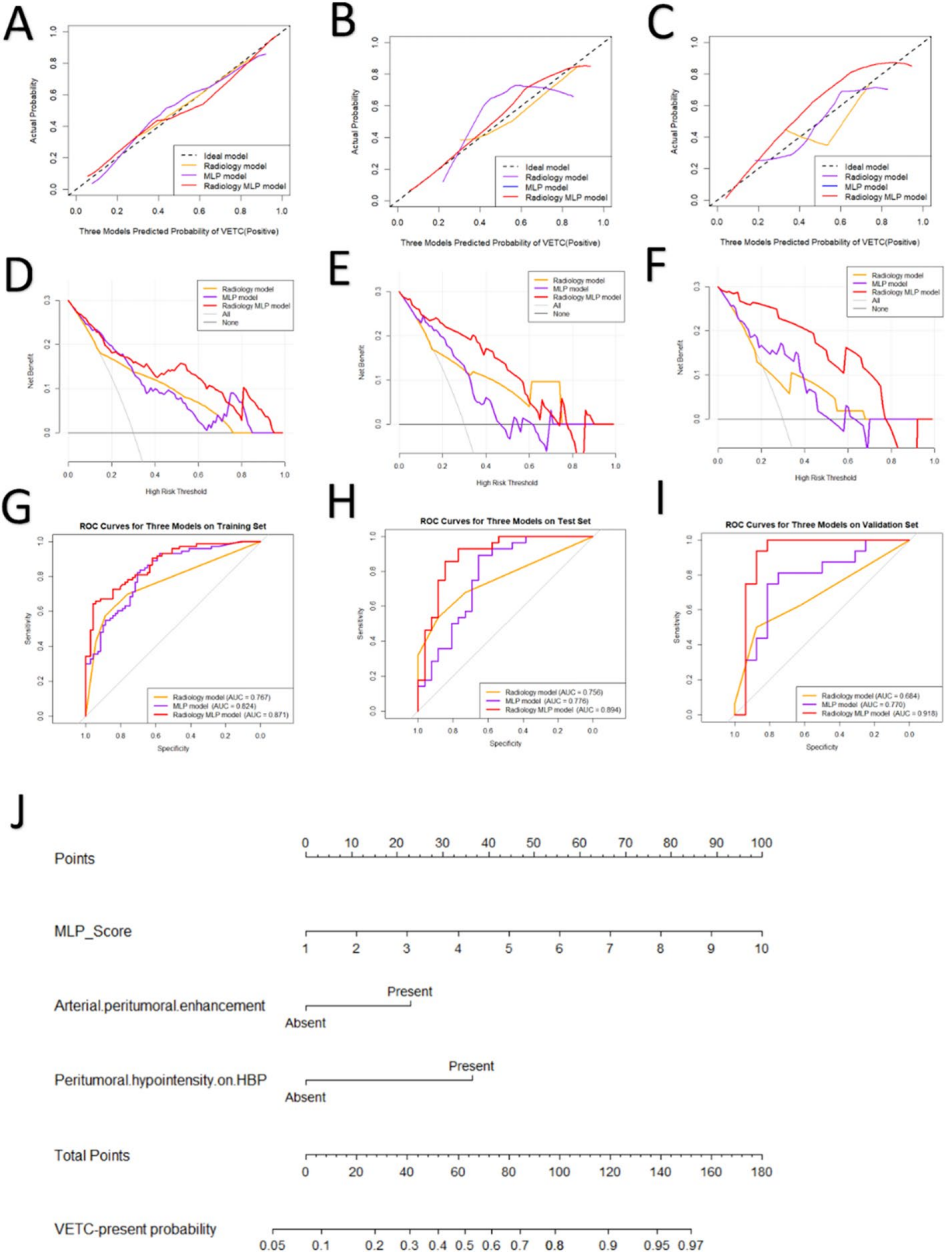
**A-I**: The calibration curve, decision curve and receiver operating characteristic (ROC) curve for three models in training, test and validation set, respectively. **J**: Nomogram of the fusion model for predicting VETC

The calibration curve, decision curve and receiver operating characteristic (ROC) curve of the radiology model, MLP model and radiology MLP model are shown in Fig. 4A-I. The radiology MLP model had a significantly higher AUC than that of MLP model and radiology model in three cohorts. There were no significant differences in AUC between the radiology model and the MLP model in three cohorts (Table 5).

**Table 5:** The comparison of performance of the three models

		Radiology vs MLP	Radiology vs Radiology MLP	MLP vs Radiology MLP
	Training	0.212	< 0.001	0.027
*P* (AUC)	Test	0.841	0.039	0.006
	Validation	0.585	0.044	0.018
	Training	0.238(0.091)	0.628(< 0.001)	0.334(0.002)
NRI (*P* value)	Test	−0.041(0.858)	0.819(< 0.001)	0.940(< 0.001)
	Validation	0.063(0.846)	1.313(< 0.001)	1.25(< 0.001)
	Training	0.061(0.208)	0.154(< 0.001)	0.093(< 0.001)
IDI (*P* value)	Test	−0.014(0.878)	0.251(< 0.001)	0.266(< 0.001)
	Validation	0.087(0.497)	0.500(< 0.001)	0.412(< 0.001)

*AUC* area under receiver operating characteristic curve, *IDI* integrated discrimination index, *NRI* net reclassification index

Delong test showed that Radiology MLP model had superior discrimination ability compared to Radiology model and MLP model. And Radiology MLP model demonstrated a 33.4%−131.3% improvement in NRI and a 9.3%−50% improvement in IDI, accordingly we select Radiology MLP model as the optimal predictive model. Additionally, the Radiology MLP model showed better calibration and clinical usefulness in three sets. The nomogram based on the Radiology MLP model is presented in Fig. 4I.

### Model explanation

**Fig. 5:**
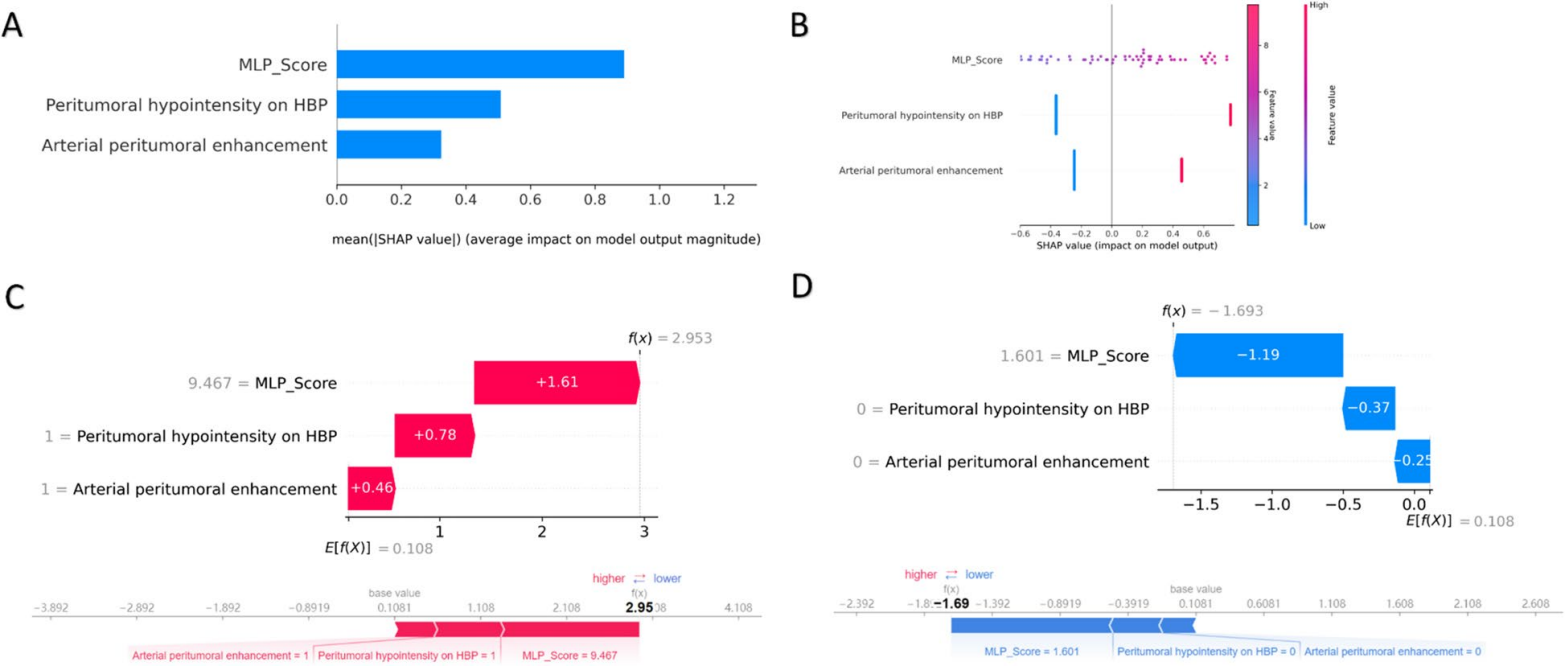
**A** Feature Importance Bar Char: displays the importance ranking of feature variables based on the radiology MLP model; **B** Summary Bee Swarm Plot: Each dot represents a single sample, with the color gradient indicating the feature value (from low to high). **C**-**D** Waterfall Plot and Force Plot: For VETC-positive patient(C), MLP-score, peritumoral hypointensity on HBP and Arterial peritumoral enhancement positively contribute to the model's prediction, with SHAP values of 1.61, 0.78 and 0.46, respectively. The total SHAP values sum up to 2.95 when added to the baseline value of 0.108, predicting the patient was VETC positive. For VETC-negative patient(D), MLP-score, peritumoral hypointensity on HBP and Arterial peritumoral enhancement negatively contribute to the model's prediction, with SHAP values of −1.19, −0.37 and −0.25, respectively. The total SHAP values sum up to −1.69 when added to the baseline value of 0.108, predicting the patient was VETC negative

We employed the SHapley Additive exPlanations (SHAP) approach for model interpretation, this method can quantify the SHAP value of each characteristic variable, representing the contribution of different factors to VETC in HCC patients. The feature importance bar chart (Fig. 5A) displays the average impact of each feature on the model output, ranked by their mean SHAP values: MLP score > Peritumoral hypointensity on HBP > Arterial peritumoral enhancement. The SHAP summary bee swarm plot (Fig. 5B) illustrates the distribution of SHAP values for each feature across all samples, showing that high values of MLP score and Peritumoral hypointensity on HBP are associated with VETC.

The SHAP waterfall plots and force plots (Figs. 5C-D) intuitively showcase the gradual contribution of individual feature on the model's prediction for VETC-positive and VETC-negative instances, showing how feature values and their corresponding SHAP values combine to form the ultimate prediction outcome.

## Discussion

In this study, we established radiology, radiomics and fusion models for preoperative prediction of VETC in HCCs based on Gd-BOPTA enhanced MRI. Our results demonstrated that the radiology MLP model showed higher performance than the radiology model and the MLP model, while the latter two models showed similar performance in the predicting VETC of HCCs. The radiology MLP model yield an incremental predictive value over the radiology model and the MLP model.

Previous study [37] had reported that VETC was significantly associated with several clinicoradiological characteristics such as arterial peritumoral enhancement and peritumoral hypointensity on HBP. In our study, the two radiology features were also independent predictors for VETC in the radiology model, which achieved an AUC of 0.756 in the test set. However, no clinical characteristics were included in the radiology model. Arterial peritumoral enhancement indicated hyperenhancement of the surrounding hepatic parenchyma. The application of Gd-BOPTA MRI to characterize HBP features offers an underutilized diagnostic pathway in HCC management.

VETC and MVI (microvascular invasion) represent critical vascular patterns implicated in hepatocellular carcinoma recurrence and metastasis. Specifically, VETC correlates with significantly worse recurrence-free survival (RFS) in HCC patients [18]. These distinct patterns reflect underlying tumor heterogeneity in metastatic mechanisms. Emerging radiological studies now aim to predict VETC status as a promising pathological biomarker [11].It was reported that more than 85% of MVI is present in the peritumoral region, [38]and that the VETC pattern is significantly associated with frequent MVI [6],which may indicate that VETC is more likely to be detected in the peritumoral region. Yu et al. previously developed intratumoral, peritumoral and combined radiomics models to indentify VETC based on Gd-BOPTA-enhanced MRI, but they just constituted combined radiomics model through combining intratumoral and peritumoral radiomics features after delineation of intratumoral region and peritumoal region, respectively. However, in our study, the ultimate fusion model based on the intratumoral and peritumoral radiomics features which were extracted as a whole ROI had a higher predictive value than other radiomics models, this was infrequent in previous studies [13, 39, 40].

In the present study, we compared seven different machine learning models within four ROIs to identify VETC of HCC patients after hepatectomy and finally determined that the MLP model was the best predictive mode of them. MLP model is a machine learning algorithm based on an artificial neural network, which can effectively analyze linear and nonlinear characteristic variables to make effective and accurate predictions [41].MLP was more suitable for small sample studies. And we further explained the radiology model through the SHAP method. SHAP is an independent machine learning model interpretation technique, which can help understand the relationship between prediction indicators and results in the radiology MLP model [42]. We applied SHAP to enhance the global interpretation of the radiology MLP model applied to the VETC prediction of HCC patients after hepatectomy and help to increase clinicians’ trust in the clinical application of the fusion radiomics model.

There were several limitations in our study. First, this paper was a retrospective study with inherent selection bias and needs to be further validated through prospective studies which is necessary to ensure the clinical applicability of the findings in our study. Second, the prognosis and overall survival analysis was not included in our study. Third, the sensitivity of the radiology MLP model in this study is relatively low.

In conclusion, the radiology MLP model performs excellently in prediction VETC of HCC patients based on based on Gd-BOPTA enhanced MRI after hepatectomy, which also yield an incremental value over the radiology and the MLP model.

## Supplementary Information

Supplementary Material 1

## Data Availability

No datasets were generated or analysed during the current study.
