# Supplementary material for: Multilayer perceptron deep learning radiomics model based on Gd-BOPTA MRI to identify vessels encapsulating tumor clusters in hepatocellular carcinoma: a multi-center study
Source: Cancer Imaging. 2025 Jul 7;25:87. doi: 10.1186/s40644-025-00895-9 (PMC12232782; doi:10.1186/s40644-025-00895-9)
Supplement: Supplementary file 1 — Supplementary Material 1 [file 40644_2025_895_MOESM1_ESM.docx]

**MRI Image Acquisition**

**Center 1:** Patients underwent MR scanning using a 1.5T scanner and an 8-channel abdominal coil (Optima MR360, GE Healthcare).

**Center 2:** Patients underwent MR scanning using a 3.0T scanner and a 16-channel abdominal coil (Discovery MR750, GE Healthcare).

**Center 3**: Patients underwent MR scanning using a 3.0T scanner and a 16-channel abdominal coil (Philips Ingenia).

**Table S1.** **Detailed scanner and scan parameters of Center 1.**

| **Sequences** | **Image plane** | **TR/TE (msec)** | **FOV (mm)** | **Flip angle** | **Thickness (mm)** | **Matrix** | **Scanning order** |
| --- | --- | --- | --- | --- | --- | --- | --- |
| T2WI | A | 6000/85 | 420 × 420 | 160 | 8 | 512 × 512 | 2 |
| T1WI | A | 190/4.3 | 420 × 420 | 80 | 8 | 256 × 160 | 1 |
| DWI | A | 3650/75 | 420 × 420 | 90 | 8 | 200 × 200 | 3 |
| AP | A | 3.7/1.7 | 420 × 420 | 15 | 5 | 256 × 200 | 4 |
| PVP | A | 3.7/1.7 | 420× 420 | 15 | 5 | 256 × 200 | 5 |
| DP | A | 3.7/1.7 | 420 ×420 | 15 | 5 | 256 × 200 | 6 |

**Table S2.** **Detailed scanner and scan parameters of Center 2.**

| **Sequences** | **Image plane** | **TR/TE (msec)** | **FOV (mm)** | **Flip angle** | **Thickness (mm)** | **Matrix** | **Scanning order** |
| --- | --- | --- | --- | --- | --- | --- | --- |
| T2WI | A | 6666/72 | 380 × 380 | 110 | 6 | 320 × 320 | 1 |
| T1WI | A | 3.7/1.7 | 400 × 400 | 12 | 6 | 512 × 512 | 2 |
| DWI | A | 7500/54 | 380 × 380 | 90 | 6 | 96 × 128 | 3 |
| AP | A | 3.7/2.2 | 360 × 360 | 12 | 5 | 512 × 512 | 4 |
| PVP | A | 3.7/2.2 | 360 × 360 | 12 | 5 | 512 × 512 | 5 |
| DP | C | 3.7/2.2 | 360 × 360 | 12 | 5 | 512 × 512 | 6 |

**Table S3.** **Detailed scanner and scan parameters of Center 3.**

| **Sequences** | **Image plane** | **TR/TE (msec)** | **FOV (mm)** | **Flip angle** | **Thickness (mm)** | **Matrix** | **Scanning order** |
| --- | --- | --- | --- | --- | --- | --- | --- |
| T2WI | A | 6400/75 | 400 × 400 | 130 | 5 | 320 × 320 | 2 |
| T1WI | A | 3.8/1.6 | 400 × 400 | 17 | 4 | 320 × 320 | 1 |
| DWI | A | 7000/60 | 400 × 400 | 90 | 5 | 128× 128 | 3 |
| AP | A | 3.8/1.6 | 400 × 400 | 17 | 4 | 320 × 320 | 4 |
| PVP | A | 3.8/1.6 | 400 × 400 | 17 | 4 | 320 × 320 | 5 |
| DP | A | 3.8/1.6 | 400 × 400 | 17 | 4 | 320 × 320 | 6 |

| **Table S4. The features of four ROI modes that selected for model construction.** |
| --- |
| **Tumor** |
| Tumor_HBP_wavelet-LHL_glcm_Idn |
| Tumor_HBP_wavelet-HLH_glszm_SmallAreaHighGrayLevelEmphasis |
| Tumor_DP_log-sigma-5-0-mm-3D_firstorder_InterquartileRange |
| **Peri2mm** |
| Peri2mm_DP_wavelet-HLL_glrlm_RunEntropy |
| Peri2mm_DWI_wavelet-LLH_gldm_SmallDependenceLowGrayLevelEmphasis |
| **Tumor+Peri2mm** |
| Tumor_HBP_wavelet-LHL_glcm_Idn |
| Tumor_DP_log-sigma-5-0-mm-3D_firstorder_InterquartileRange |
| Peri2mm_DP_wavelet-HLL_glrlm_RunEntropy |
| **TumorPeri2mm** |
| TumorPeri2mm_DP_wavelet-LHL_glcm_MaximumProbability |
| TumorPeri2mm_HBP_original_gldm_DependenceNonUniformityNormalized |
| TumorPeri2mm_DWI_wavelet-HLH_firstorder_Kurtosis |
|  |


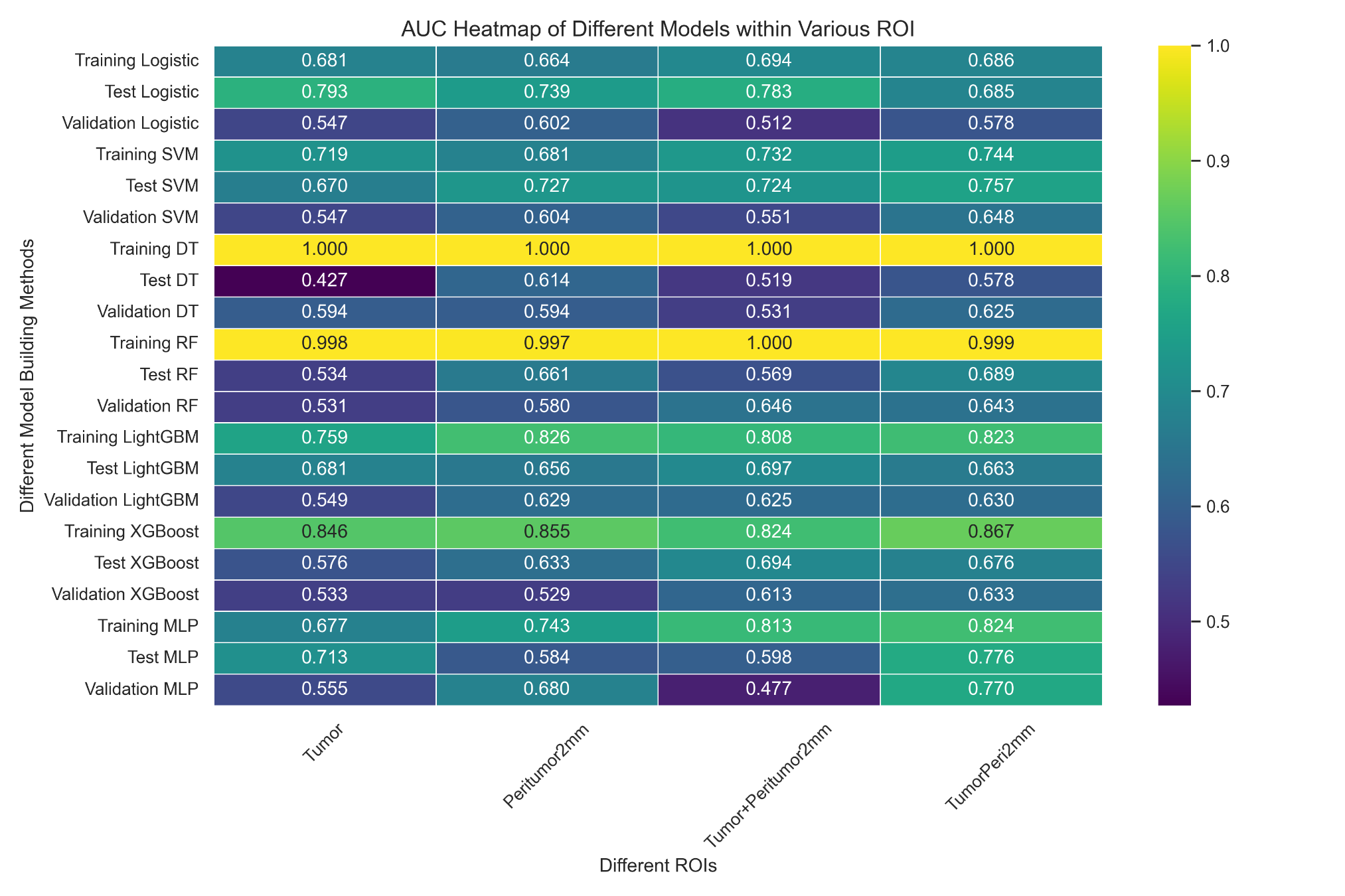


**Fig S1. Heatmap depicting the AUCs between different models within various ROI.**


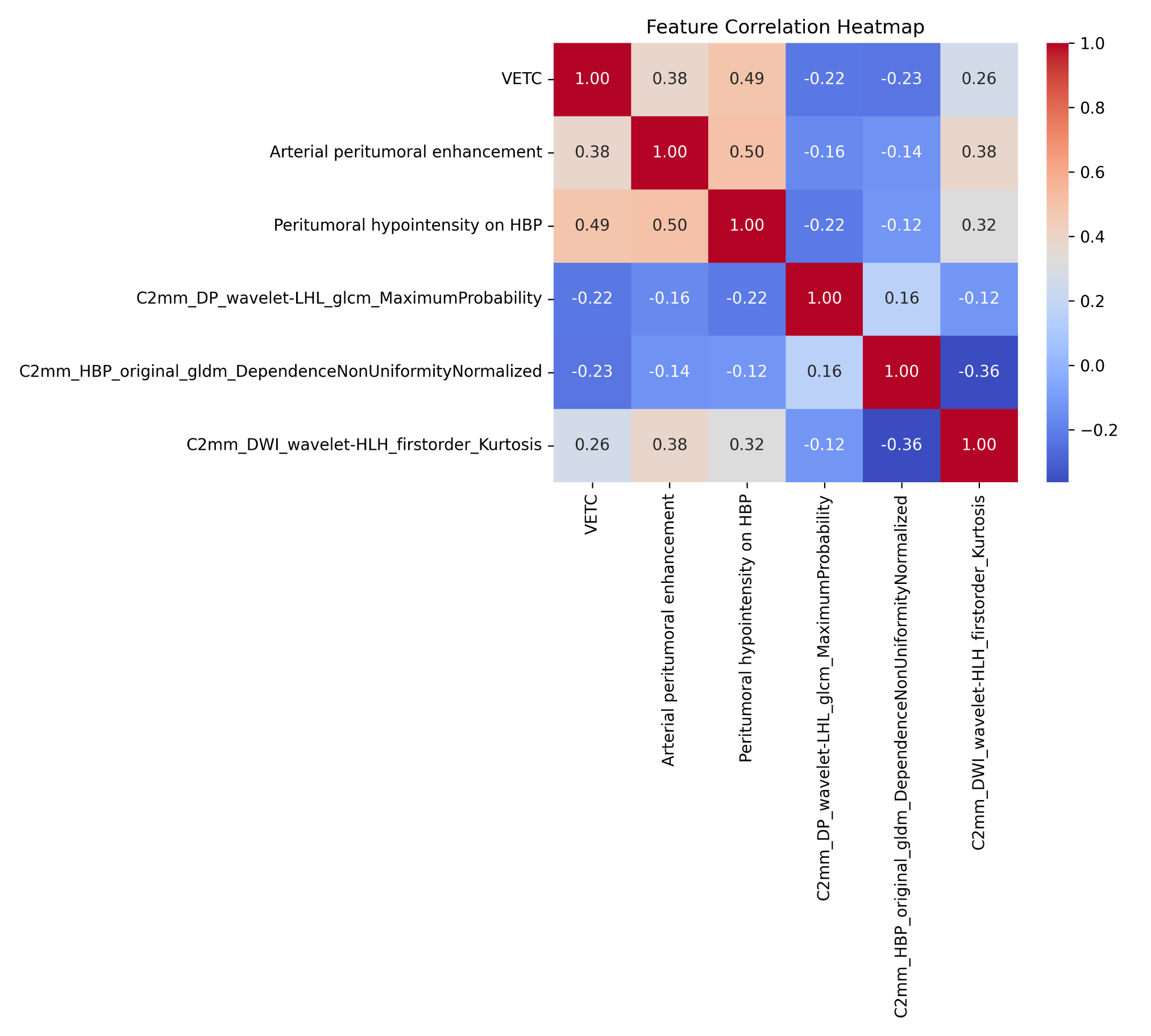


**Fig S2. Heatmap depicting correlation coefficients matrix of 5 selected features in the selected radiology and radiomics features.**
